# Exploring a causal role of DNA methylation in the relationship between maternal vitamin B_12_ during pregnancy and child’s IQ at age 8, cognitive performance and educational attainment: a two-step Mendelian randomization study

**DOI:** 10.1093/hmg/ddx164

**Published:** 2017-04-27

**Authors:** Doretta Caramaschi, Gemma C. Sharp, Ellen A. Nohr, Katie Berryman, Sarah J. Lewis, George Davey Smith, Caroline L. Relton

**Affiliations:** 1 Medical Research Council Integrative Epidemiology Unit, School of Social and Community Medicine; 2 School of Oral and Dental Sciences, The Cleft Collective, University of Bristol, Bristol BS8 2BN, UK; 3 Research Unit for Gynaecology and Obstetrics, Department of Clinical Research, University of Southern Denmark, Odense 5000C, Denmark; 4 School of Social and Community Medicine, University of Bristol, Bristol BS8 2BN, UK

## Abstract

An adequate intake of vitamin B_12_ during pregnancy plays an important role in offspring neurodevelopment, potentially via epigenetic processes. We used a two-step Mendelian randomization approach to assess whether DNA methylation plays a mediating and causal role in associations between maternal vitamin B_12_ status and offspring’s cognition. Firstly, we estimated the causal effect of maternal vitamin B_12_ levels on cord blood DNA methylation using the maternal *FUT2* genotypes rs492602:A > G and rs1047781:A > T as proxies for circulating vitamin B_12_ levels in the Avon Longitudinal Study of Parents and Children (ALSPAC) and we tested the observed associations in a replication cohort. Secondly, we estimated the causal effect of DNA methylation on IQ using the offspring genotype at sites close to the methylated CpG site as a proxy for DNA methylation in ALSPAC and in a replication sample. The first step Mendelian randomization estimated that maternal vitamin B_12_ had a small causal effect on DNA methylation in offspring at three CpG sites, which was replicated for one of the sites. The second step Mendelian randomization found weak evidence of a causal effect of DNA methylation at two of these sites on childhood performance IQ which was replicated for one of the sites. The findings support a causal effect of maternal vitamin B_12_ levels on cord blood DNA methylation, and a causal effect of vitamin B_12_-responsive DNA methylation changes on children’s cognition. Some limitations were identified and future studies using a similar approach should aim to overcome such issues.

## Introduction

Components of one carbon metabolism, which includes folate, and several B vitamins, play an important role in prenatal nutrition and have been implicated in a range of neurodevelopmental disorders in offspring (1). Periconceptional folic acid or multivitamin supplementation has been shown to protect offspring against neural tube defects (2–6) and more recently folic acid supplementation during pregnancy has been linked to reduced risk of severe language delay (7) and autism in children (8). Adequate circulating vitamin B_12_ levels during pregnancy are also associated with a decreased risk of neural tube defects (9) and there is evidence that maternal vitamin B_12_ status during pregnancy is associated with offspring cognition (10–13).

The mechanisms through which sub-optimal levels of circulating vitamin B_12_ exert unfavourable effects during pregnancy are not fully understood. One possible pathway is via the role of vitamin B_12_ in one carbon metabolism and the donation of methyl groups for the methylation of a range of biological molecules including DNA. Profound changes in DNA methylation which occur in early development in the brain and nervous system may be particularly sensitive to vitamin B_12_ availability during foetal life. Emerging evidence suggests that maternal prenatal vitamin B_12_ status influences DNA methylation at the insulin-like growth factor-II locus and at a global level (14–16).

A recurring problem in addressing questions of maternal prenatal nutrition and offspring outcomes is distinguishing causal relationships in the face of multiple potential confounding factors. A Mendelian randomization (MR) approach has been widely applied in an attempt to strengthen causal inference and circumvent the issue of confounding in such studies (13,17–23). In a Mendelian randomization framework, a genetic variant is used as a proxy for the exposure of interest. This approach can be implemented when considering vitamin B_12_ as an exposure, as genetic variants have recently been reported that are robustly associated with vitamin B_12_ levels. For example, genome-wide association studies have identified common genetic variants in the *FUT2* gene to be associated with serum vitamin B_12_ levels in individuals with European ancestry (24–27) and in a Chinese population (28). Two *FUT2* SNPs that are strongly associated with vitamin B_12_ levels are rs492602:A > G and rs1047781:A > T. In a previous study (13), maternal genotype at the common single nucleotide polymorphism (SNP) rs492602:A > G in the *FUT2* gene was found to be associated with offspring’s IQ at age 8. Since maternal *FUT2* genotype is not associated with socioeconomic confounders that might affect maternal vitamin B_12_ levels and intake, these findings suggest that maternal vitamin B_12_ levels are causally related to children’s IQ but have little effect. These findings were not confirmed in more recent studies (29,30) and the intermediate effect of DNA methylation has not been previously examined.

**Figure 1:**
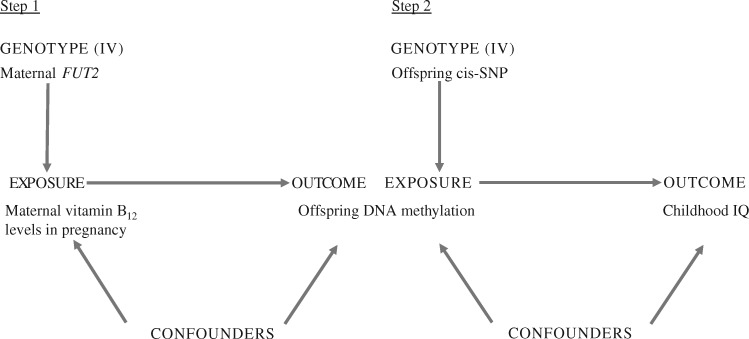
Diagram showing the two-step Mendelian randomization approach used in this study. Step 1: The maternal *FUT2* genotype is used as instrumental variable (IV) for circulating vitamin B_12_ levels in pregnancy (exposure) as its effect on cord blood CpG methylation (outcome) is not vulnerable to confounders. Step 2: The offspring genotype at a cis-SNP around the CpG whose methylation is affected by maternal *FUT2* genotype is used as IV for CpG methylation to estimate the effect of cord blood methylation (exposure) on childhood IQ (outcome) as it is free from confounder biases.

An extension of Mendelian randomization to investigate mediation has been described (17,31), and the particular application to epigenetic studies elaborated upon (17). The aim of this method is to assess whether DNA methylation plays a mediating and causal role in linking an exposure to an outcome. In ‘two-step epigenetic Mendelian randomization’ a SNP is used to proxy for the exposure of interest but rather than explore its association with the phenotype of interest (here child IQ), DNA methylation is considered as an outcome in the first step. If DNA methylation variation is associated with the exposure, then a second Mendelian randomization step can be applied using an SNP that proxies for methylation levels at the site modified by the exposure of interest, and its association with the interrogated exposure. The process is summarised in Figure 1. The rationale for this study is twofold. Firstly, based on indications in the literature that maternal vitamin B_12_ levels during pregnancy affect cognitive neurodevelopment, we wanted to address the biological question of whether epigenetic changes play a role in this association. Secondly, we wanted to explore and develop the recently established Mendelian randomization methodology and apply it to a novel setting within the context of mediation.

In this study, we investigated the potential mediating role of DNA methylation in the observed relationship between maternal prenatal vitamin B_12_ levels and offspring IQ using a two-step epigenetic Mendelian randomization (MR) approach (17). In the first step, we used maternal genetic variants at the *FUT2* gene as proxies for *in utero* vitamin B_12_ exposure on offspring DNA methylation at the genome-wide level. Site-specific differences in DNA methylation (CpG sites) identified in step 1 were taken forward to step 2; in the second step we used offspring SNPs near the CpGs of interest as proxies for offspring DNA methylation, and used these to estimate the causal effect of DNA methylation in children on their own IQ. If DNA methylation mediates an association between prenatal vitamin B_12_ exposure and IQ, we should observe firstly that the maternal *FUT2* genetic variation is associated with offspring DNA methylation and secondly that SNPs that proxy for the associated methylation sites are associated with childhood IQ. The SNP effect on both the exposure and the outcome might indicate causality or a pleiotropic effect (the outcome and the exposure are affected by the same SNP) as elucidated recently (22,32,33). Ideally, the outcomes of both MR steps should be justified a-priori or based on analyses in a separate dataset to avoid bias. However, because available datasets are lacking and no previous studies have investigated DNA methylation as a mediator on the causal pathway between maternal *FUT2* genotype and childhood IQ, we performed both steps in subgroups of the same study population and tried to replicate the first and second step in separate studies. This work has also highlighted other challenges associated with two-step Mendelian randomization that future studies should consider and attempt to overcome.

## Results

**Figure 2:**
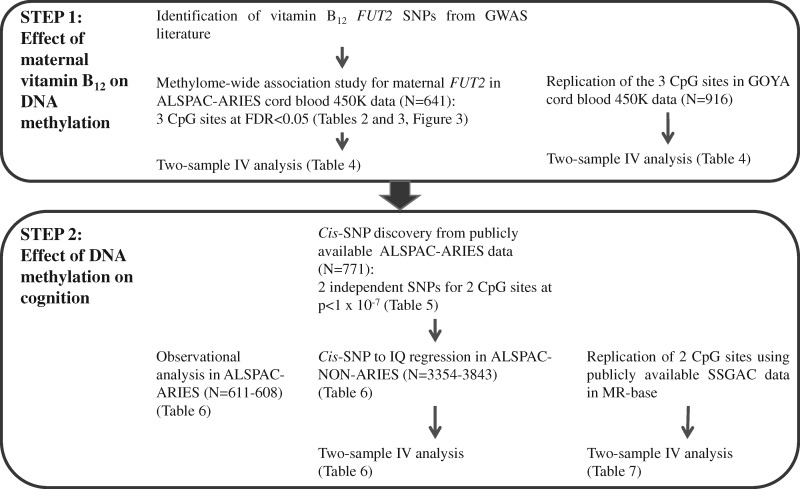
Flow diagram showing the two steps of the analysis conducted.

A flow-chart summarizing the two-steps diagram including the datasets used and relevant results tables is presented in Figure 2.

### Study sample characteristics

We performed the first and second step MR in the Avon Longitudinal Study of Parents and Children (ALSPAC) study (34,35). From the original eligible sample, only subsets of subjects had the data needed for each analysis step (mother and child genetic, epigenetic, IQ). The numbers of subjects included in each different analysis are shown in the corresponding result tables. Table 1 shows the characteristics of the study population and of the subsamples used in the 1^st^–step MR and the 2^nd^–step MR. The children that were participants in the first step MR analysis had slightly older and better educated mothers who smoked less than the children in the second step MR analysis. The children of the first step MR analysis also were tested at a slightly younger age on average and had higher IQ scores. Mother’s BMI, parity and child’s sex did not seem to differ between the two study samples.

**Table 1:** Characteristics of study populations in the ALSPAC sub-samples used in the first step MR and second step MR analyses and in the GOYA sample

	ALSPAC (ARIES) (First step MR)			ALSPAC (NON-ARIES) (Second step MR)			*P*-value^a^	GOYA (First step)		
Continuous variables	Mean	SD	N	Mean	SD	N		Mean	SD	N
Mother’s age at delivery (years)	29.33	4.36	641	29.19	4.57	3843	0.003	29.58	4.01	916
Mother’s BMI	22.78	0.14	641	22.93	0.06	3501	0.314	29.41	7.64	916
Child’s age at testing (years)	8.62	0.18	641	8.67	0.28	3501	<0.001	–	–	–
Total IQ	107.69	15.62	568	104.44	16.42	3843	<0.001	–	–	–
Verbal IQ	110.82	16.63	571	107.49	16.60	3843	0.005	–	–	–
Performance IQ	101.94	16.81	571	99.83	16.94	3843	<0.001	–	–	–
**Categorical variables**	**%**		**N**	**%**		**N**		**%**		**N**
Mother’s education (university level)	22.15		641	16.96		3095	0.001	49.78		916
Child’s sex (male)	47.41		599	49.36		3843	0.375	51.31		916
Parity (previous pregnancies)	51.17		641	53.12		3761	0.360	53.06		916
Smoking during pregnancy	12.79		641	18.42		3779	0.001	22.27		916

a *P*-values for ALSPAC (ARIES) vs ALSPAC non-ARIES were calculated using Student’s t test for continuous variables and χ^2^ test for categorical variables.

BMI, body mass index.

The replication of the first step was carried out in the Genetics of Overweight Young Adults (GOYA) study. The GOYA study was similar to the ALSPAC in terms of age of delivery, child’s sex and parity. However, BMI was higher, and there was a higher proportion of pregnancy smokers and highly educated mothers. The characteristics of the study populations are summarized in Table 1.

**Figure 3:**
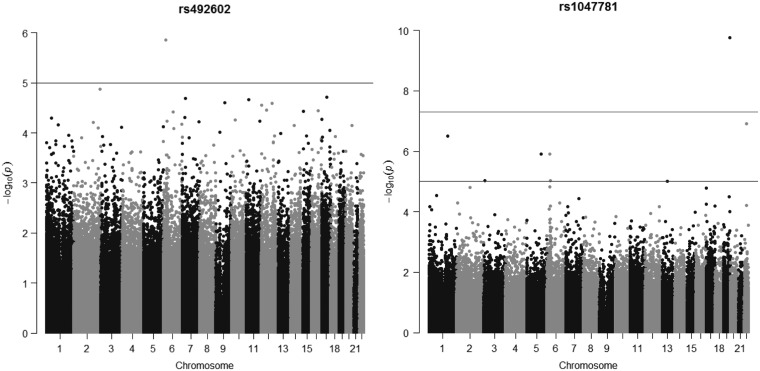
Manhattan plots showing the results of the methylome-wide association study (MWAS) for maternal *FUT2* genotype in cord blood in the Avon Longitudinal Study of Parents and Children (ALSPAC), adjusted for batch, cell composition, child’s genotype, mother’s body mass index (BMI), mother’s education, mother’s age at conception, smoking during pregnancy and parity.

*1^st^‒step MR.* The association of maternal *FUT2* genotype with cord blood DNA methylation was explored by running a methylome-wide association study (MWAS) in ALSPAC. The results are presented in Figure 3 and the top 20 CpGs with the lowest *P*-values are reported in Tables 2 and 3. The MWAS for mother’s rs492602:A > G genotype (genomic inflation factor λ = 0.969) did not show any CpG site where offspring DNA methylation was associated with the maternal *FUT2* genotype at FDR ≤ 0.05. The rs1047781:A > T MWAS (λ = 0.802) revealed three CpGs where offspring DNA methylation was associated with the maternal *FUT2* genotype at FDR ≤ 0.05. Maternal *FUT2* genotype was associated with lower DNA methylation at cg23332223 in an intergenic region upstream the gene *USP29* and it was associated with higher methylation at cg10543947 and cg15676719, at the transcription start sites of the genes *APOL2* and *RCSD1*. These three CpG sites were carried forward in the first step MR analysis.

**Table 2:** Association of CpG methylation and maternal *FUT2* rs492602:A > G genotype (*n =* 641)

CpG	β^a^	S.E.^a^	*P*-value	N	FDR	Bonferroni	Gene	Gene region	Chr.	Position
cg00468410	0.010	0.002	1.40E-06	604	0.656	0.656			6	20239552
cg10979567	−0.007	0.001	1.35E-05	594	1	1	COL6A3	Body	2	238241642
cg21877220	0.002	0.000	1.96E-05	598	1	1			17	46695449
cg19701577	−0.005	0.001	2.03E-05	589	1	1	HOXA5	3'UTR	7	27181418
cg08835103	0.002	0.000	2.15E-05	594	1	1	DBX1	Body	11	20178138
cg13895650	−0.034	0.008	2.47E-05	606	1	1	TLE1	Body	9	84228185
cg10998227	0.013	0.003	2.56E-05	603	1	1			12	101108809
cg11808677	0.007	0.002	2.79E-05	597	1	1	USP5	TSS1500	12	6960079
							CDCA3	Body		
cg04053798	−0.014	0.003	3.55E-05	606	1	1	SLC38A4	1^st^ exon	12	47219705
								5'UTR		
cg11933375	0.002	0.000	3.63E-05	590	1	1	SLC38A7	5'UTR	16	58718466
cg10601234	0.007	0.002	3.67E-05	592	1	1			15	25523666
cg03993171	0.001	0.000	3.83E-05	598	1	1	SNORD50B	TSS1500	6	86388501
							SNORD50A	TSS200		
							SNHG5			
cg18116968	0.019	0.005	4.99E-05	604	1	1			7	25900668
cg00256155	0.002	0.001	5.12E-05	599	1	1			1	47915647
cg18222500	0.028	0.007	5.41E-05	604	1	1	RPH3AL	Body	17	143285
cg06223926	−0.023	0.006	5.55E-05	606	1	1			10	33626522
cg27049594	0.054	0.013	5.77E-05	606	1	1	OR8A1	TSS1500	11	124439146
cg24508713	0.017	0.004	5.79E-05	603	1	1	ZBTB12	TSS1500	6	31870783
cg07470532	−0.017	0.004	6.08E-05	602	1	1			7	153442389
cg06571387	0.008	0.002	6.24E-05	604	1	1	HOXD12	TSS1500	2	176964101

a β coefficients and standard errors for association tests are calculated using linear regression with number of minor alleles at the rs492602:A > G SNP as independent variable and CpG proportion methylation as dependent variable (beta-values), adjusted for offspring’s genotype, batch (surrogate variables), maternal age, maternal body mass index, maternal smoking, maternal education, parity and estimated cell counts. The β coefficient is to be interpreted as the change in methylation proportion per minor-allele count unit increase.

FDR, false discovery rate; Chr., chromosome.

**Table 3:** Association of CpG methylation and maternal *FUT2* rs1047781:A > T genotype (*n =* 641)

CpG	β^a^	S.E.^a^	*P*-value	N	FDR	Bonferroni	Gene	Gene region	Chr.	Position
cg23332223	−0.259	0.040	1.71E-10	606	8.03E-05	8.03E-05			19	57626946
cg10543947	0.241	0.045	1.22E-07	606	0.029	0.057	APOL2	TSS200, 1^st^ exon, 5'UTR	22	36635882
cg15676719	0.170	0.033	3.18E-07	606	0.050	0.149	RCSD1	TSS1500	1	167598521
cg10886493	0.161	0.033	1.21E-06	548	0.113	0.565	HLA-A	Body	6	29911036
cg11290181	−0.100	0.020	1.21E-06	593	0.113	0.566	CDC42SE2	5'UTR	5	130604045
cg10466124	0.045	0.010	9.46E-06	597	0.574	1	HLA-DRB5	TSS1500	6	32498285
cg14992144	0.059	0.013	9.46E-06	600	0.574	1	GHRLOS	Body	3	10334743
							GHRL	TSS200		
cg21480902	0.057	0.013	9.80E-06	606	0.574	1			13	68682181
cg13582692	−0.270	0.062	1.50E-05	606	0.703	1	NCRNA00171	Body	6	30022577
cg25868126	−0.081	0.019	1.58E-05	584	0.703	1	TMEM177	3'UTR	2	120439606
cg08489349	0.187	0.043	1.65E-05	606	0.703	1	ELP2P	Body	17	656181
							GEMIN4	TSS1500		
cg14830466	−0.025	0.006	2.91E-05	603	1	1	CTH	TSS1500	1	70876729
cg08194323	0.111	0.027	3.18E-05	606	1	1	NAPSA	Body	19	50862004
cg16913250	0.192	0.046	3.61E-05	584	1	1	CTTNBP2	TSS1500	7	117513835
cg04549115	0.015	0.004	5.06E-05	598	1	1	RWDD1	TSS200	6	116892534
cg24201793	0.095	0.023	5.08E-05	605	1	1	MBOAT2	TSS1500	2	9144764
cg17624832	−0.028	0.007	5.56E-05	593	1	1	NXN	Body	17	862284
cg16121206	0.245	0.061	6.06E-05	606	1	1	APOL2	TSS200	22	36636055
cg20503907	0.096	0.024	6.40E-05	606	1	1	MICB	Body	6	31474086
cg04934595	−0.015	0.004	6.43E-05	585	1	1			17	81021419

a β coefficients and standard errors for association tests are calculated using linear regression with number of minor (T) alleles at the rs1047781:A > T SNP as independent variable and CpG proportion methylation as dependent variable (beta-values), adjusted for offspring’s genotype, batch (surrogate variables), maternal age, maternal body mass index, maternal smoking, maternal education, parity and estimated cell counts. The β coefficient is to be interpreted as the change in methylation proportion per minor-allele count unit increase.

FDR, false discovery rate; Chr., chromosome.

The two-sample MR analysis for the first step was performed using the genotype-exposure estimates from the GWAS study on vitamin B_12_ levels previously published by Lin *et al.* (28). Specifically, beta coefficient = 70.21 (pg/ml) and standard error = 5.53 (pg/ml) were used. The results are reported in Table 4. In line with the above results, maternal vitamin B_12_ levels were associated with decreased cord blood methylation upstream the gene *USP29*, and with higher cord blood methylation at *APOL2* and *RCSD1*. Our analysis estimated that 1 pg/ml higher maternal vitamin B_12_ increased or decreased cord blood methylation by less than 1% at each site investigated.

**Table 4:** Step 1: IV estimates of the causal effect of maternal vitamin B_12_ levels on cord blood CpG methylation

		ALSPAC (N = 641)			GOYA (N = 916)		
CPG Label	Gene	IV estimate^a^	S.E.	*P*-value	IV estimate^a^	S.E.	*P*-value
cg23332223		−0.004	0.0006	7.17 x 10^−9^	−0.0008	0.0003	0.006
cg10543947	*APOL2*	0.003	0.0007	7.97 x 10^−7^	0.0006	0.0006	0.358
cg15676719	*RCSD1*	0.002	0.0005	1.66 x 10^−6^	0.0005	0.0002	0.038

a Change in proportion methylation (unit is 100%) per one unit increase in maternal plasma vitamin B_12_ (pg/ml).

IV, instrumental variable.

In the GOYA cohort, the association of maternal *FUT2* genotype at rs1047781:A > T was in the same direction for all the three CpGs investigated. There was evidence of replication for cg23332223 (*P*-value < Bonferroni 0.05) as shown in Table 4.

*2^nd^‒step MR.* The second step MR was carried out in the ALSPAC and using summary data from the Social Science Genetic Association Consortium (SSGAC) (36–38). In order to perform the second step MR we looked in the ARIES mQTL database for cis-SNPs for the vitamin B_12_-responsive CpG sites discovered in the first step. At birth, there were no SNPs associated with cg23332223 DNA methylation, therefore we could not perform the second step MR for this CpG site. There were six SNPs associated with DNA methylation at cg10543947, which were not independent and, amongst these, only rs5750236:C > T had data in the SSGAC database, therefore we used rs5750236:C > T as IV in the 2^nd^-step MR for this CpG. For cg15676719, 91 SNPs were associated with DNA methylation, and there was only one independent cis-SNP, rs1890131:C > T, which was therefore taken forward as IV in the 2nd step MR. The characteristics of the chosen cis-SNP are reported in Table 5.

**Table 5:** Independent cis-SNPs selected in ARIES as instrumental variable for *FUT2*-responsive CpG methylation in the 2^nd^-step MR

CpG	Gene	Chr.	SNP	MAF	SNP Position	β^a^	S.E.	*P*-value
cg10543947	*APOL2*	22	rs5750236:C>T	0.4312	36585162	−0.26	0.04	7.28 x 10^−09^
cg15676719	*RCSD1*	1	rs1890131:C>T	0.4044	167597473	−0.44	0.04	1.36 x 10^−25^

a The β coefficient is to be interpreted as the change in methylation proportion (unit is 100%) per minor allele count unit increase.

MR, Mendelian randomization; Chr., chromosome, MAF; minor allele frequency.

Table 6 shows the results of the associations of cis-SNP genotype and DNA methylation at corresponding CpG sites with children’s IQ in the ALSPAC sample after excluding the samples with methylation data as they were used for the discovery of cis-SNPs associations with methylation. The estimates from the conventional (non-IV) analyses between DNA methylation and IQ had very large standard errors and high *P*-values. However, using MR, there was some evidence for a minimal causal effect of DNA methylation on increasing performance IQ at *APOL2* and decreasing overall IQ and performance IQ at *RCSD1*.

**Table 6:** Step 2: Association of DNA methylation with IQ at age 8

CpG site	Gene	SNP	Outcome	SNP effect (N = 3354 -3843)^a^	*P*-value	Methylation effect (observational) (N = 611–608)^b^	*P*-value	Methylation effect (IV) (N = 3354 –3843)^c^	*P*-value	Vitamin B_12_ effect via CpG methylation (10 pg/ml)^d^
cg10543947	*APOL2*	rs5750236:C>T	Overall IQ	−0.53 (0.40)	0.19	−1.17 (7.36)	0.87	2.03 (1.58)	0.20	0.06
			Verbal IQ	−0.13 (0.41)	0.75	−0.51 (7.71)	0.95	0.49 (1.57)	0.75	0.01
			Performance IQ	−0.91 (0.42)	0.03	−0.44 (8.21)	0.96	3.46 (1.71)	0.04	0.10
cg15676719	*RCSD1*	rs1890131:C>T	Overall IQ	0.82 (0.38)	0.03	1.59 (10.92)	0.88	−1.87 (0.88)	0.03	−0.03
			Verbal IQ	0.53 (0.38)	0.16	−4.86 (11.42)	0.67	−1.23 (0.88)	0.17	−0.02
			Performance IQ	0.99 (0.39)	0.01	8.91 (12.16)	0.46	−2.26 (0.92)	0.01	−0.04

a β coefficients (S.E.) indicate the change in IQ score units per minor allele count.

b β coefficients (S.E.) indicate the change in IQ score units per methylation proportion (unit is 100%).

c IV estimates (S.E.) represent the change in IQ score units per methylation proportion (unit is 100%).

d The effect of vitamin B_12_ on IQ score is calculated by multiplying the IV estimates of the second step MR by the IV estimates of the first step MR and it is to be interpreted as the change in IQ score units per 10 pg/ml maternal vitamin B_12_ increase via DNA methylation at the individual CpG site.

IV, instrumental variable.

The two-sample analysis conducted in MR-base using the SSGAC database (Table 7) provided some evidence for an increase in childhood intelligence via DNA methylation at the *APOL2* gene. Neither methylation site was associated with cognitive/educational outcomes in the larger study. Childhood intelligence data for *RCSD1* were not available.

**Table 7:** IV estimates of the effect of DNA methylation at birth on cognition and educational attainment

CpG site	SNP	Outcome	N	β^a^	S.E.^a^	*P*-value
cg10543947	rs5750236:C>T	Childhood intelligence	12,441	0.145	0.062	0.019
	rs5750236:C>T	Cognitive performance	106,736	−0.007	0.020	0.740
	rs5750236:C>T	College completion	126,559	−0.025	0.046	0.592
	rs5750236:C>T	Years of schooling	126,559	−0.006	0.015	0.689
cg15676719^b^	rs1890131:C>T	Cognitive performance	106,736	−0.016	0.010	0.110
	rs1890131:C>T	College completion	126,559	−0.007	0.023	0.765
	rs1890131:C>T	Years of schooling	126,559	−0.014	0.009	0.134

a βs and standard errors (S.E.) were calculated using the online tool MR-base which used data from the ARIES project (genotype-methylation) and the SSGAC consortium (methylation-outcome) and are to be interpreted as a change in cognitive performance, college completion and years of schooling (standardized scores) per proportion methylation unit.

b Childhood intelligence data were not available for this SNP.

IV, instrumental variable.

### Functional characterization of the methylation sites

The relatively novel *USP29* gene codes for a ubiquitin-specific processing protease and it is involved in ubiquitin-dependent protein catabolic processes and thiol-dependent ubiquitinyl hydrolase activity. Some evidence show a role in the repair of DNA damage and cell survival (39). It is expressed mainly in the testis, while virtually no expression was found in the brain. An association with urate levels in European lean men is reported in the GWAS Catalog (40). The *APOL2* gene encodes for a cytoplasmatic protein that is involved in lipid transport and lipoprotein metabolic processes. It is expressed in most tissues, including the brain and the pituitary gland. It is upregulated in the prefrontal cortex of schizophrenic patients (41), and the GWAS Catalog reports an association with non-diabetic end-stage renal disease in African Americans (42). The *RCSD1* gene encodes for a protein involved in actin filament binding. It was found to be expressed at minimal levels in the brain, while it was highly expressed in EBV-transformed lymphocytes, lung, muscle, small intestine, spleen and whole blood. No associations were found in OMIM or in the GWAS Catalog.

### Hypothesis-free MR analysis of other potential health outcomes

Within MR-Base we performed a hypothesis-free MR analysis of the association of DNA methylation with the disease outcomes. For the *APOL2* variant there were 32 diseases with available genotype. The analysis showed weak evidence of an association with type 2 diabetes (β = 0.11, S.E.=0.06, uncorrected *P*-value = 0.047) and coronary heart disease (β=−0.15, S.E.=0.08, uncorrected *P*-value = 0.05). For the *RCSD1* variant there were 32 diseases with available genotype. The analysis revealed some association with ulcerative colitis (β = 0.14, S.E.=0.05, uncorrected *P*-value = 0.008) and inflammatory bowel disease (β = 0.10, S.E.=0.04, uncorrected *P*-value = 0.018). The full results are in Supplementary Materials, Table S1 and S2.

## Discussion

Increasing evidence suggests that DNA methylation is an intermediate link between prenatal malnutrition and offspring’s neurocognitive development (1,9,10,12,29,43,44). We used a two-step Mendelian randomization (MR) approach to investigate whether prenatal exposure to maternal vitamin B_12_ levels is causally linked to offspring’s IQ at age 8 via changes in offspring’s DNA methylation. In the first MR step, we used maternal genotype in the *FUT2* gene at rs492602:A > G and at rs1047781:A > T two genetic variants that are strongly associated with vitamin B_12_ levels to estimate the causal association with DNA methylation. Our IV analysis suggests that maternal vitamin B_12_ status is causally associated with small differences in DNA methylation in the cord blood of offspring. In the second MR step, we used the offspring genotype at cis-SNPs associated with DNA methylation at the three CpG sites most strongly associated with maternal vitamin B_12_ (identified in the first step) and estimated the causal association between DNA methylation and IQ. The causal estimates for this association were positive at one site and negative at another site, therefore not providing enough evidence for a positive effect of increasing vitamin B_12_ levels prenatally on offspring intelligence. Some caution is warranted because the CpG sites taken forward to the second step were selected amongst those that were associated with the instrument from the first step (maternal *FUT2* genotype), therefore a causal effect on DNA methylation was expected for these sites and the causal effect estimates for the first step might be overestimated through this procedure. The replication cohort provided more evidence for the associations found in the first step, at least for one site, but we could not take forward this site to the second step. Moreover, the effect sizes were much smaller than in the discovery cohort. We tried to replicate the findings of the second step using two-sample MR in MR-base where we used GWAS summary data on childhood intelligence, cognitive performance and years of schooling and we replicated a positive effect of DNA methylation at the *APOL2* gene on childhood intelligence, while we could not replicate the negative effect at the *RCSD1* gene. Overall, our study suggests a small positive effect of prenatal vitamin B_12_ on child’s IQ through DNA methylation. Moreover, DNA methylation at vitamin B_12_-responsive genes seemed to have an effect on other disease outcomes as ulcerative colitis and irritable bowel disease, although these results need further investigation. The approach taken in this study highlighted several other shortcomings that are discussed below and should be considered when performing two-step Mendelian randomization in future studies.

A possible confounder in Mendelian randomization is population stratification, however the ALSPAC study is mostly representative of white mother-child pairs since non-white mothers constitute only 2.2% of the ALSPAC sample (33) and genotyping and epigenotyping has been undertaken on mainly white mother-child pairs. Moreover, we excluded mothers and children that were classified “non-white”. The drawback of this approach is that the results are not generalizable to non-white or more mixed populations.

One typical limitation to consider in Mendelian randomization studies is often the low power due to a small genetic effect on the exposure. This is even more of a consideration in two step MR where numbers need to be an order of magnitude greater to account for the power needed at each stage. In the first step, our study had 80% power to detect at true effect size f^2^=0.01 of maternal *FUT2* on DNA methylation at α = 0.05/485000 and *n =* 641. The first step two-sample analysis in ALSPAC had 80% power to detect a true standardized causal effect β = 0.52 at *R*^2^=0.05, α = 0.05 and *n =* 641 (online calculator https://sb452.shinyapps.io/power/; date last accessed May 03, 2017) (45). Our findings in ALSPAC were still largely underpowered due to the low minor allele frequency of *FUT2* genotype and due to the small effect sizes therefore we tried to overcome this by running the 1^st^ step MR in a separate cohort, where we had 80% power to detect a true effect β = 0.42 at *R*^2^=0.05, α = 0.05 and *n =* 916. Since we focused our investigation on the top CpG sites resulting from the methylome-wide association analysis, we may have missed sites with weaker association with *FUT2* that could potentially still be associated with IQ. The 450K data set contains probes for only around 1% of the CpGs that are potentially methylated in the whole genome. However, more informative technologies such as sequencing are not yet affordable for large sample sizes from population studies. In the second step, we had 80% power to detect cis-SNPs for cord blood DNA methylation at a true effect size f^2^=0.01 at α = 0.05/6000000 and *n =* 770. Using those cis-SNPs in the two-sample MR allowed us to detect true standardized causal effects β = 0.45 at 80% power, α = 0.05 and *n =* 3843. Therefore, we tested the 2^nd^ step MR additionally in a larger consortium, where we had 80% power to detect a causal effect β = 0.025 at α = 0.05 and *n =* 12441. Moreover, only two out of three CpG sites had independent SNPs and therefore there is a need to identify *cis*-SNPs in larger datasets.

Another limitation of Mendelian randomization relevant to our study is the potential for confounding due to pleiotropy and linkage disequilibrium (LD). An on-line search on the GWAS Catalog (46) has revealed that *FUT2* is associated with phenotypes such as obesity-related traits, Crohn’s disease, cholesterol levels and bipolar disorder. These phenotypes might be downstream to the effects on vitamin B_12_ levels (vertical pleiotropy) and therefore they might not represent a problem in the interpretation of the Mendelian randomization analysis. However, as the molecular biology of *FUT2* is still unclear in relation to vitamin B_12_, we cannot exclude potential biological pathways that do not involve vitamin B_12_ (horizontal pleiotropy). A hypothesis-free look-up in MR-base of all available traits and outcomes identified associations of rs492602:A > G *FUT2* genotype with Crohn’s disease, cholesterol levels, inflammatory bowel disease and type 1 diabetes, but no associations for rs1047781:A > T *FUT2* genotype, the SNP on which our findings are based. A future study could combine multiple genetic variants to proxy for vitamin B_12_ in order to overcome pleiotropy. In the second step, we used independent cis-SNPs for methylation, as these may be less likely to have pleiotropic effects and LD-induced confounding than trans-SNPs. However, the children’s genotype at the methylation sites showed some weak association with phenotypes that are also affected by *FUT2*, suggesting some potential pleiotropy perhaps in relation to their own *FUT2* genotype.

Although blood is relatively easy to access in large population studies, epigenetic marks are often tissue-specific, so stronger associations might have been found in central nervous system tissue. Although this is a potential limitation, obvious ethical and logistical reasons precluded us from using such tissue in this study and the relevance of these findings in blood must be carefully considered. Some studies have suggested little overlap between blood and brain DNA methylation patterns (47,48) and some associations between blood DNA methylation and brain-related processes, for instance with the functionality of the serotonin pathway (49), childhood physical aggressiveness (50), major depressive disorder (51), autistic spectrum disorder (52) and schizophrenia (53). However, the concordance is highly heterogeneous between different loci and can be minimal (54–56). Regarding our specific findings, a publicly available dataset (http://epigenetics.essex.ac.uk/bloodbrain/; date last accessed May 03, 2017) (47) show some correlation between adult brain and blood in two out of the three vitamin-B_12_ responsive CpGs (Supplementary Material, Fig. S1), the sites that we took forward in step 2 MR (for cg15676719 the correlation is for one brain area only). At this stage, it is still not possible to know whether blood and brain correspondence translates into similar effects from the maternal exposure to vitamin B_12_ and this issue clearly requires further investigation.

Finally, the two-sample IV approach can be cost-effective, especially when gene-exposure association estimates from large GWAS studies are available (45). However, it assumes that the two independent samples are comparable. The published study we used is based on Chinese men. The SNP rs1047781:A > T used as IV for prenatal vitamin B_12_ exposure seems to be a specific SNP for Chinese populations (MAF = 0.459) and it is rare in the ARIES (MAF = 0.004) and in the GOYA mothers (MAF= .009) that participated in this study. The difference in the MAF between the two samples might create a bias in the IV estimate. Data on the association between vitamin B_12_ and *FUT2* genotype in pregnant women are lacking and there is to date no indication that hormonal changes affect the association between *FUT2* genotype and vitamin B_12_ levels. In a small subset of ALSPAC we found evidence for a positive association only for child’s rs492602:A > G genotype, (Supplementary Material, Table S1) and it remained after adjusting for maternal genotype. Furthermore, although there is evidence for a correlation between maternal and cord vitamin B_12_ status (57), there is also evidence suggesting that maternal vitamin B_12_ deficiency might not translate into a foetal vitamin B_12_ deficiency (58). Consequently, impaired vitamin B_12_ levels in the mothers might affect foetal DNA methylation through mechanisms other than lowering foetal vitamin B_12_ levels that have yet to be explored.

To our knowledge, this is the first report of a methylome-wide study of the effects of maternal prenatal vitamin B_12_ on cord blood DNA methylation. Previous studies showed that maternal vitamin B_12_ levels were associated with cord blood hypomethylation of the promoter of the *IGF2* gene (15) and overall hypomethylation of genomic DNA (14). Another study found no association with cord blood LINE-1 methylation (16). Other studies have found that maternal levels of other components of the one-carbon metabolism affect cord blood DNA methylation (43,59). Together with these other studies, our data suggest that during pregnancy an adequate intake of micronutrients involved in one-carbon pathway could affect DNA methylation. With regards to cognitive development, the association found in our study with *APOL2* methylation in the blood might reflect changes in lipid transport and metabolism in the brain that in turn alter brain functioning and cognition (60). The hypothesis-free analysis performed to extend the question to a wider range of health consequences suggests causal effects beyond the cognitive phenotype.

In conclusion, we applied a two-step Mendelian randomization approach to investigate DNA methylation as a mediator between a prenatal exposure and a childhood outcome. We found some evidence of a causal effect of maternal vitamin B_12_ levels on cord blood DNA methylation and little evidence of a causal effect of DNA methylation on childhood intelligence. This work has highlighted the various strengths and particular challenges associated with two-step Mendelian randomization and we hope that it will motivate further use of the approach in similar contexts where DNA methylation is hypothesised to mediate observed associations between a prenatal exposure and a later-life phenotype.

## Materials and Methods

### ALSPAC data

The subjects for the main part of this study were participants of the ALSPAC (see for details (34,35). The participants included in the study were from the core sample, singletons, and of white ethnicity. Please note that the study website contains details of all the data that is available through a fully searchable data dictionary (http://www.bris.ac.uk/alspac/researchers/data-access/data-dictionary/; date last accessed May 03, 2017). Ethical approval for the study was obtained from the ALSPAC Ethics and Law Committee and the Local Research Ethics Committees. Mothers’ and children’s genetic data at specified SNPs (see below) were extracted from the ALSPAC GWAS database (34). Briefly, the genetic data for mothers (*FUT2* genotype) were generated using the Illumina human660W-quad and the IlluminaGenomeStudio calling algorithm. Quality Control measures included the removal of SNPs with more than 5% of missingness, a Hardy-Weinberg-Equilibrium *P*-value lower than 10^−6^ and a minor allele frequency on less than 1%. Samples with more than 5% missingness, indeterminate X chromosome heterozygosity or extreme autosomal heterozygosity were excluded. SNP imputation was carried out against the 1000 Genome Project database (www.1000genomes.org; date last accessed May 03, 2017) (61). Genetic data for the children were generated by Sample Logistics and Genotyping Facilities at the Wellcome Trust Sanger Institute and LabCorp (Laboratory Corportation of America) using support from 23andMe using the Illumina Human Hap 550-quad and the Illumina GenomeStudio calling algorithm. Quality control and imputation were performed as above.

Neonatal blood DNA methylation data were generated using the IlluminaHumanMethylation450 BeadChip as part of the ARIES project (http://www.ariesepigenomics.org.uk; date last accessed May 03, 2017 (62)), where DNA methylation was measured in white cells extracted from cord blood (82% of the subjects) and in cord blood spots (18% of the subjects) and using the Illumina Infinium 450K platform. Briefly, DNA samples were bisulphite converted using the Zymo EZ DNA methylationTM kit (Zymo, Irvine, CA). Following conversion genome-wide methylation was measured using the Illumina HumanMethylation450 (HM450K) BeadChip. The arrays were scanned using an Illumina iScan, with initial quality review using GenomeStudio. For each sample the proportion of DNA molecules methylated at each CpG site is represented as a beta-value (63). Samples with >20% probes with *P*-value  > = 0.01 were excluded from further analysis and scheduled for repeat assay. Genotype probes on the HM450k were compared between samples from the same individual and against SNP-chip data to identify and remove any sample mismatches. Methylation data were normalised with the wateRmelon R package (64), using the “Tost” algorithm to reduce the non-biological differences between probes (65). All the processing of the methylation data was done using the meffil R package (available at https://github.com/perishky/meffil; date last accessed May 03, 2017).

After excluding multiple births, genetic data were available for *n =* 8890 mothers and *n =* 8871 children whereas epigenetic data were available for *n =* 914 children. The complete-cases sample containing genetic (mother and children), epigenetic and covariate data used for the MWAS (first step MR) was *n =* 641.

Childhood cognition was measured as the overall intelligence score (IQ) derived from a shortened version of the Wechsler Intelligence Scale for Children (WISC-III) that was administered to the children at approximately 8 years of age (66,67). The Verbal IQ score was computed using the age-scaled scores obtained by administering the Information, Similarities, Arithmetic, Vocabulary and Comprehension subtests. The Performance IQ score is composed of the age-scaled scores of Picture Completion, Coding, Picture Arrangement, Block Design and Object Assembly subtests. IQ data were available from *n =* 5164 children. Amongst these, *n =* 611 participants had epigenetic data and the relevant covariates and were used for the conventional (non-IV analysis), whereas *n =* 3843 that did not include the ones with epigenetic data were used for the IV analysis (second step MR).

### Replication data

The subjects for the replication study of the first step MR were participants in the GOYA Study which is described previously by Paternoster *et al.* (68). It is based on the Danish National Birth Cohort (69) and included 2451 women that were pregnant during 1996–2002, had given birth to a live born infant, had provided a blood sample during pregnancy and had a BMI that ranged from 32.6 to 64.4 and 2450 pregnant women during the same period that were chosen at random. Of these, 1,960 extremely overweight and 1,948 control women were genotyped using the Illumina Human610-Quad v1.0 BeadChip and passed quality control. Cord blood DNA methylation data were generated for the offspring of 1000 mothers in the GOYA study. Cord blood was collected according to standard procedures, spun and frozen at −80˚C. DNA methylation analysis and data pre-processing were performed at the University of Bristol using the same protocol as for the generation of the ARIES (ALSPAC) data. The complete-case sample was *n =* 916.

The replication of the second step MR was carried out using summary data from published studies that were carried out by the Social Science Genetic Association Consortium (SSGAC) (36–38).

### Two-step MR analyses

Two-step Mendelian randomization analysis (Fig. 1) was performed using instrumental variable (IV) analysis. For each step, the IV estimator was calculated as the ratio between the β coefficient of the genotype-outcome regression and the β coefficient of the genotype-exposure regression. The standard error was calculated as reported in Thomas *et al.,* 2007 (70).

*1^st^‒step MR: Examining the causal effect of maternal vitamin B_12_* *o* *n DNA methylation.* We first evaluated the association between the genotype-outcome (*FUT2*-methylation) association in the ALSPAC-ARIES study by conducting a methylome-wide association study (MWAS) using the CpGassoc R package (71). Briefly, after removing probes on sex chromosomes and probes with *P*-value > 0.05, and after removing outliers, a linear regression model measured the additive effect of the minor allele in the maternal genotype at the rs492602:A > G SNP and at the rs1047781:A > T in the *FUT2* gene on DNA methylation at 468,622 CpG sites in cord blood. Batch (10 surrogate variables), cell composition estimated for cord blood as in Bakulski *et al.,* (72), child’s genotype, mother’s BMI, mother’s education, mother’s age at conception, smoking during pregnancy and parity were included in the model as covariates as they could potentially influence DNA methylation at birth and therefore they could potentially lead to errors in the phenotype assessment. The three CpG sites observed to be most strongly associated with maternal *FUT2* based on the MWAS (false discovery rate, FDR, ≤0.05) were taken forward in the IV analysis, which estimated the causal exposure-outcome relationship, i.e. the causal effect of maternal vitamin B_12_ levels on offspring DNA methylation. We then tested the top three *FUT2*-CpG associations using the genetic and epigenetic data from the GOYA cohort. For this analysis, we followed the same samples and probes exclusions and statistical models as for the ALSPAC cohort, including the same covariates.

Functional analyses for the methylation genome-wide significance hits were carried out using online tools. A search for functional annotation terms was performed using the Gene Ontology Consortium website (www.geneontology.org; date last accessed May 03, 2017) (73). Gene expression was examined in the Genotype-Tissue Expression (GTEx) portal version V6p (www.gtexportal.org; date last accessed May 03, 2017). A literature search for potential associations with health phenotypes was performed using the NHGRI-EBI GWAS Catalog of published genome-wide association studies (www.ebi.ac.uk/gwas/; date last accessed May 03, 2017) (46) and the Online Mendelian Inheritance in Man (OMIM) database (www.omim.org; date last accessed May 03, 2017).

*2^nd^‒step MR: Examining the causal effect of DNA methylation on IQ.* Firstly, conventional (non-IV) linear regression analysis was performed to assess the association between DNA methylation (the exposure) and IQ (the outcome) in the ALSPAC cohort. These analyses were carried out using beta-values for each of the CpG sites identified as associated with maternal *FUT2* in step 1. Models were adjusted for age of testing batch (surrogate variables), mother’s BMI, mother’s age at delivery, mother’s education, smoking during pregnancy, and parity.

Next, Mendelian randomization was used to estimate the causal association between DNA methylation and IQ. For the methylation probes discovered in step 1, cis-SNPs were identified in the mQTL database (http://mqtldb.org; date last accessed May 03, 2017) (74), which contains the associations between CPG methylation and SNPs observed in the ARIES project at *P* < 10^−7^. These were SNPs associated with CpG methylation and were located within 1 Mb either side of the CpG. Independent cis-SNPs, obtained through LD clumping, were selected as the instrumental variables for CpG methylation and the β coefficient and standard error for the cis-SNP-methylation association were used in the IV analysis. The sample size for this analysis was *n =* 770.

For the genotype-outcome associations, IQ was regressed on the cis-SNPs in the ALSPAC cohort, excluding participants that were used to identify cis-SNPs, as it has been shown that choosing variants according to their strength of association with exposure in the data under analysis can give biased estimates (75,76). The models included age of testing as a covariate.

The 2^nd^‒step MR was also run in a separate study in order to investigate replication and benefit from the increased power of a larger study sample. A two-sample MR was carried out using MRbase (http://www.mrbase.org/beta/; date last accessed May 03, 2017), an online platform that contains data from 985 genome-wide association studies and interfaces with the R package TwoSampleMR (https://github.com/MRCIEU/TwoSampleMR; date last accessed May 03, 2017). Cognitive and education genome-wide association data were available from the Social Science Genetic Association Consortium (SSGAC) (36–38). We selected childhood intelligence, cognitive performance, college completion and years of schooling as outcomes.

## Supplementary Material

Supplementary Material is available at *HMG* online.

## Supplementary Material

Supplementary Data
