# Supplementary material for: Exploring a causal role of DNA methylation in the relationship between maternal vitamin B12 during pregnancy and child’s IQ at age 8, cognitive performance and educational attainment: a two-step Mendelian randomization study
Source: Hum Mol Genet. 2017 Apr 27;26(15):3001–13. doi: 10.1093/hmg/ddx164 (PMC5703349; doi:10.1093/hmg/ddx164)
Supplement: Supplementary Data [file ddx164_supp.docx]

**Table S1.** Association of DNA methylation at cg10543947 with other diseases calculated using hypothesis-free IV analysis in MRbase.

| **Disease** | **Beta** | **S. E.** | **p-value** |
| --- | --- | --- | --- |
| Type 2 diabetes \|\| diagram \|\| 2014 | 0.112781 | 0.056715 | 0.046751 |
| CoroNAry heart disease \|\| CARDIoGRAM \|\| 2011 | -0.15432 | 0.078729 | 0.049987 |
| Parkinson's disease \|\| dbGAP \|\| 2009 | 0.25815 | 0.156213 | 0.098423 |
| Type 2 diabetes \|\| diagramplusmetabochip \|\| 2012 | 0.082833 | 0.055668 | 0.136756 |
| Myocardial infarction \|\| CARDIoGRAMplusC4D \|\| 2015 | -0.05689 | 0.038967 | 0.144291 |
| Ulcerative colitis \|\| IIBDGC \|\| 2011 | -0.11278 | 0.085519 | 0.18724 |
| Rheumatoid Arthritis trans ethnic \|\| Okada \|\| 2014 | 0.077083 | 0.059611 | 0.195974 |
| CoroNAry heart disease \|\| CARDIoGRAMplusC4D \|\| 2013 | -0.05609 | 0.044019 | 0.202576 |
| Neuroblastoma \|\| dbGAP \|\| 2013 | 0.195709 | 0.164519 | 0.23421 |
| Anorexia Nervosa \|\| GCAN \|\| 2014 | -0.131 | 0.117143 | 0.263442 |
| Lung cancer (squamous cell) \|\| ILCCO \|\| 2014 | -0.1079 | 0.101011 | 0.285437 |
| Multiple sclerosis \|\| dbGAP \|\| 2009 | -0.2633 | 0.250633 | 0.293478 |
| ischaemic stroke \|\| dbGAP \|\| 2007 | 1.146886 | 1.138055 | 0.31357 |
| Rheumatoid Arthritis Asian \|\| Okada \|\| 2014 | 0.116217 | 0.120567 | 0.335087 |
| Ulcerative colitis (Euro) \|\| IIBDGC \|\| 2015 | -0.07402 | 0.080507 | 0.357856 |
| Rheumatoid Arthritis Euro \|\| Okada \|\| 2014 | 0.077083 | 0.087804 | 0.38 |
| Systemic lupus erythematosus \|\| dbGAP \|\| 2008 | 0.143059 | 0.175615 | 0.415294 |
| Lung cancer (all) \|\| ILCCO \|\| 2014 | -0.05217 | 0.066904 | 0.435499 |
| schizophrenia \|\| dbGAP \|\| NA | 0.003194 | 0.006652 | 0.6311 |
| asthma \|\| gabriel \|\| 2007 | -0.03797 | 0.079145 | 0.631447 |
| Inflammatory Bowel Disease (Euro) \|\| IIBDGC \|\| 2015 | -0.02555 | 0.063719 | 0.688384 |
| CoroNAry heart disease (additive) \|\| CARDIoGRAMplusC4D \|\| 2015 | -0.00897 | 0.035358 | 0.799814 |
| Rheumatoid arthritis \|\| Stahl \|\| 2010 | -0.03797 | 0.163405 | 0.816275 |
| Alzheimer's disease \|\| IGAP \|\| 2013 | 0.009157 | 0.05914 | 0.876948 |
| CKD \|\| CKDGen \|\| 2015 | -0.00878 | 0.061048 | 0.885698 |
| CoroNAry heart disease \|\| C4D \|\| 2011 | -0.00572 | 0.0641 | 0.928855 |
| Crohn's disease (Euro) \|\| IIBDGC \|\| 2015 | -0.00496 | 0.086993 | 0.954561 |
| Eczema \|\| EAGLE \|\| 2015 | -0.00315 | 0.064312 | 0.9609 |
| Lung adenocarcinoma \|\| ILCCO \|\| 2014 | -0.00309 | 0.10356 | 0.976183 |
| bipolar disorder \|\| dbGAP \|\| 2009 | 0 | 0.129241 | 1 |
| familial Parkinson disease \|\| dbGAP \|\| 2011 | 0 | 0.016408 | 1 |
| inflammatory bowel disease \|\| dbGAP \|\| 2006 | 0 | 0.066856 | 1 |

**Table S2.** Association of DNA methylation at cg15676719 with other diseases calculated using hypothesis-free IV analysis in MRBase.

| **Disease** | **Beta** | **S.E.** | **p-value** |
| --- | --- | --- | --- |
| Ulcerative colitis (Euro) \|\| IIBDGC \|\| 2015 | 0.136184 | 0.051439 | 0.008109 |
| Inflammatory Bowel Disease (Euro) \|\| IIBDGC \|\| 2015 | 0.096686 | 0.040875 | 0.018011 |
| Lung cancer (squamous cell) \|\| ILCCO \|\| 2014 | 0.122584 | 0.0627 | 0.050572 |
| ischaemic stroke \|\| dbGAP \|\| 2007 | 1.290833 | 0.791546 | 0.102938 |
| Lung adenocarcinoma \|\| ILCCO \|\| 2014 | -0.09328 | 0.069472 | 0.179369 |
| Type 2 diabetes \|\| diagram \|\| 2014 | 0.045474 | 0.034134 | 0.182792 |
| Alzheimer's disease \|\| IGAP \|\| 2013 | -0.04593 | 0.038809 | 0.236638 |
| Rheumatoid Arthritis Asian \|\| Okada \|\| 2014 | -0.06788 | 0.062928 | 0.280737 |
| Crohn's disease (Euro) \|\| IIBDGC \|\| 2015 | 0.059246 | 0.056031 | 0.29034 |
| CoroNAry heart disease \|\| CARDIoGRAM \|\| 2011 | 0.031362 | 0.033019 | 0.342209 |
| CKD \|\| CKDGen \|\| 2015 | 0.029853 | 0.036742 | 0.416505 |
| Rheumatoid arthritis \|\| Stahl \|\| 2010 | -0.02285 | 0.040417 | 0.571834 |
| Rheumatoid Arthritis trans ethnic \|\| Okada \|\| 2014 | -0.02285 | 0.040819 | 0.575631 |
| CoroNAry heart disease (additive) \|\| CARDIoGRAMplusC4D \|\| 2015 | 0.012465 | 0.023396 | 0.594191 |
| Eczema \|\| EAGLE \|\| 2015 | 0.021567 | 0.04052 | 0.594544 |
| Myocardial infarction \|\| CARDIoGRAMplusC4D \|\| 2015 | 0.002991 | 0.02604 | 0.908549 |
| Lung cancer (all) \|\| ILCCO \|\| 2014 | -0.00355 | 0.043466 | 0.934827 |

**Table S3**. Association of maternal and children’s *FUT2* genotype with vitamin B_12_ levels in cord blood and in offspring’s blood at age 7, unadjusted and mutually adjusted for each other’s genotype.

|  | **rs492602** | | | | | | **rs1047781** | | | | | |
| --- | --- | --- | --- | --- | --- | --- | --- | --- | --- | --- | --- | --- |
|  | **Cord blood** | | | **Age 7** | | | **Cord blood** | | | **Age 7** | | |
|  | **N** | **Beta (S.E.)** | **P-value** | **N** | **Beta (S.E.)** | **P-value** | **N** | **Beta (S.E.)** | **P-value** | **N** | **Beta (S.E.)** | **P-value** |
| Mother | 250 | 0.03 (0.05) | 0.57 | 250 | 0.05 (0.03) | 0.04 | 250 | -0.59 (0.31) | 0.06 | 250 | 0.06 (0.19) | 0.76 |
| Child | 296 | 0.02 (0.04) | <0.0005 | 294 | 0.15 (0.03) | <0.0005 | 296 | -1.05 (0.31) | 0.001 | 294 | 0.02 (0.20) | 0.92 |
| Mother adjusted for child | 239 | -0.11 (0.05) | 0.04 | 240 | -0.01 (0.03) | 0.68 | 239 | 0.30 (0.62) | 0.62 | 240 | 0.23 (0.39) | 0.56 |
| Child adjusted for mother | 239 | 0.28 (0.05) | <0.0005 | 240 | -0.15 (0.03) | <0.0005 | 239 | -1.08 (0.67) | 0.11 | 240 | -0.02 (0.41) | 0.62 |

Betas and P-values are obtained from linear regression of *FUT2* allele dosage on ln-transformed vitamin B_12_ levels.

**Figure S1**

**
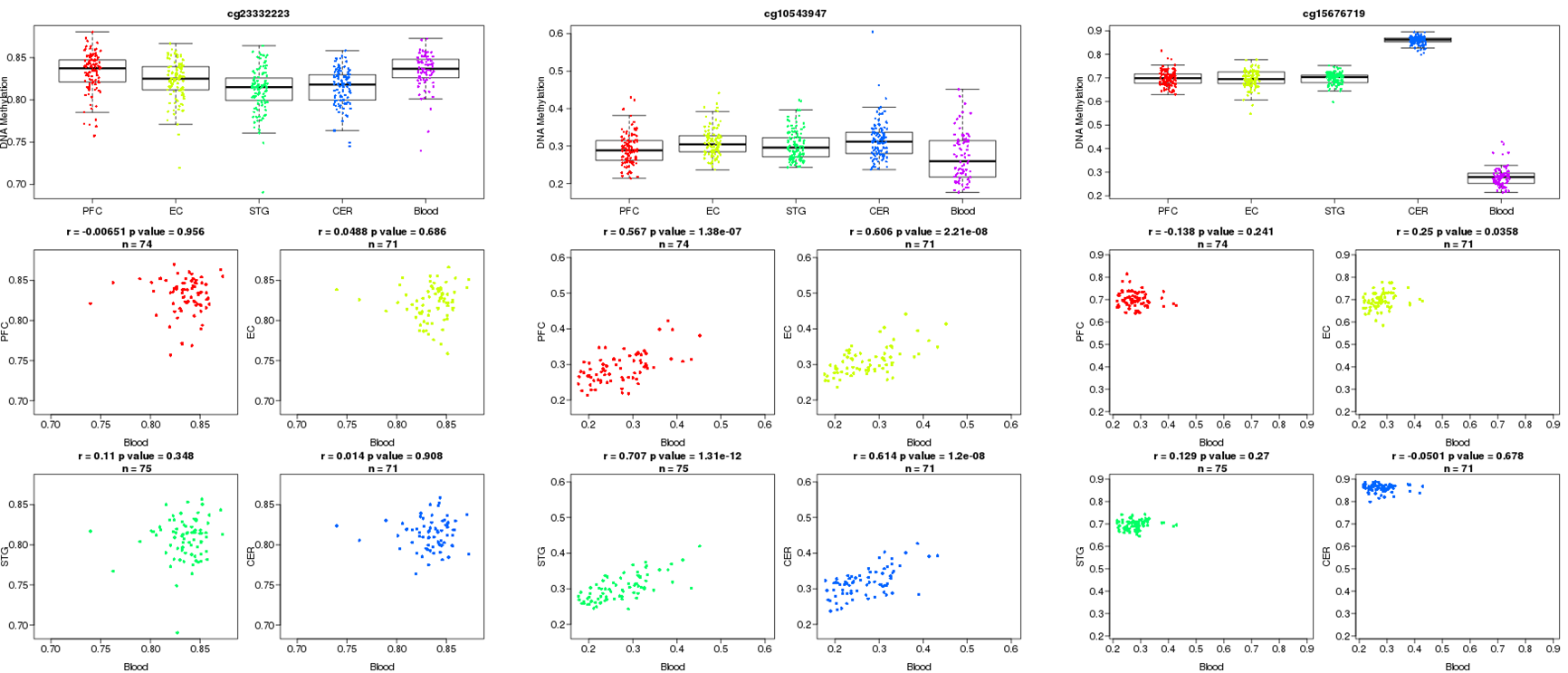
**

**Figure S1.**

DNA methylation levels in the blood and in brain of adult individuals (N=75) at the vitamin B_12_-responsive CpGs. For each CpG, the top panel shows the actual methylation values and the four panels below show the correlation between blood and each brain area investigated. DNA methylation was measured by Illumina Infinium 450K and the data and the graphs were obtained from <http://epigenetics.essex.ac.uk/bloodbrain/> (1).

**References**

1. Davies MN, Volta M, Pidsley R, Lunnon K, Dixit A, Lovestone S, et al. Functional annotation of the human brain methylome identifies tissue-specific epigenetic variation across brain and blood. Genome Biol 2012;13:R43.
